# Carrier cross-reactivities of the bile acid reabsorption inhibitors elobixibat, linerixibat, maralixibat, and odevixibat

**DOI:** 10.1016/j.jlr.2025.100910

**Published:** 2025-09-23

**Authors:** Veronica Billo, Christopher Neelen, Marie Wannowius, Anita Neubauer, Bärbel Fühler, Yohannes Hagos, Joachim Geyer

**Affiliations:** 1 Institute of Pharmacology and Toxicology, Faculty of Veterinary Medicine, Biomedical Research Center Seltersberg (BFS), Justus Liebig University of Giessen, Giessen, Germany; 2 PortaCellTec Biosciences GmbH, Göttingen, Germany

**Keywords:** SLC10, SOAT, NTCP, OATP, bile acid reabsorption inhibitor, SC-435, bile acids and salts, transport, drug therapy, liver

## Abstract

The bile acid reabsorption inhibitors (BARIs) elobixibat, maralixibat, and odevixibat are clinically used inhibitors of the intestinal bile acid transporter ASBT (*SLC10A2*). An additional BARI compound, linerixibat, is still under clinical development. In the present study, potential cross-reactivities against the closely related hepatic bile acid carrier and hepatitis B virus entry receptor NTCP (*SLC10A1*), as well as the steroid sulfate uptake carrier SOAT (*SLC10A6*) were analyzed. All BARIs potently inhibited ASBT (IC_50_ = 0.1–1.0 μM). Among them, elobixibat, maralixibat, and odevixibat also inhibited SOAT (IC_50_ = 3.2–5.9 μM) and NTCP (IC_50_ = 10–99 μM). Furthermore, all four BARIs inhibited the hepatic drug transporters OATP1B1, OATP1B3, and OATP2B1 (IC_50_ = 1.6–29 μM). Notably, ASBT inhibition by linerixibat was reversible upon washout, while maralixibat and odevixibat induced full and sustained ASBT inhibition even after removal of the inhibitor and inhibitor-free incubation over 240 min. Elobixibat and the pan-SLC10 inhibitor troglitazone revealed an intermediate effect. The ASBT S294T/I295V double mutation increased the inhibitory potency of linerixibat, suggesting a role of this domain for linerixibat binding. In contrast, this mutation had no significant effect on the ASBT inhibition by elobixibat, maralixibat, and odevixibat, indicating distinct binding sites. In conclusion, the analyzed BARIs revealed carrier cross-reactivities with NTCP, SOAT, and members of the OATP family, but behaved differently regarding their time-dependent inhibition and potential inhibitor binding sites.

## Graphical Abstract

**Figure:**
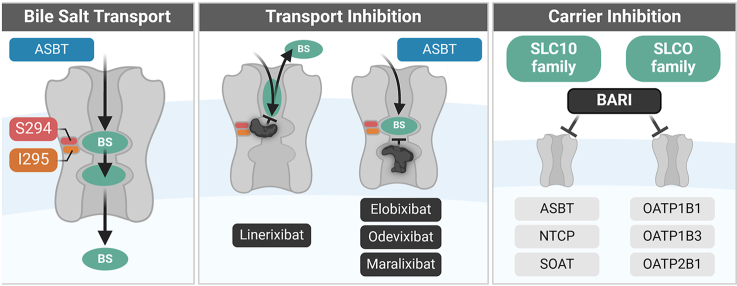

The enterohepatic circulation of bile salts (BS) is an efficient recycling process and involves several active transport systems in the liver and in the gut (Fig. 1) (1, 2, 3). BS are synthesized from cholesterol in hepatocytes, involving the action of 16 different enzymes (Fig. 1A) (2) and then are actively secreted into bile by the bile salt efflux pump BSEP (gene symbol *ABCB11*) that is located at the canalicular membrane of hepatocytes (Fig. 1B) (4). With the bile flow, BS are excreted into the intestinal lumen, where they form micelles and facilitate absorption of dietary lipids and fat-soluble vitamins (5). At the terminal ileum, about 99% of the intestinal BS are actively reabsorbed via the apical sodium-dependent bile acid transporter ASBT (gene symbol *SLC10A2*). This carrier belongs to the solute carrier family SLC10 and is localized at the apical brush border membrane of enterocytes (Fig. 1C) (6). The heteromeric organic solute transporter OSTα-OSTβ then is responsible for BS efflux at the basolateral membrane of enterocytes (7). With the portal blood flow, the reabsorbed BS are transported back to the liver, where they are actively taken up via the Na^+^/taurocholate co-transporting polypeptide NTCP (gene symbol *SLC10A1*) that is localized at the basolateral membrane of hepatocytes (Fig. 1D) (8). In addition, carriers from the organic anion transporting polypeptide (OATP) family contribute to the hepatic uptake of BS. Within hepatocytes, BS can downregulate their de novo synthesis via a feedback mechanism, involving farnesoid X receptor (FXR) activation and down-regulation of the rate-limiting enzyme cytochrome P450 CYP7A1 (Fig. 1A) (2, 5, 9). In humans, a pool of about 1.5–4.0 g of BS circulates about 6–10 times a day between the liver and the intestine and about 17–40 g of BS are actively transported via ASBT in the small intestine per day (10).

**Fig. 1:**
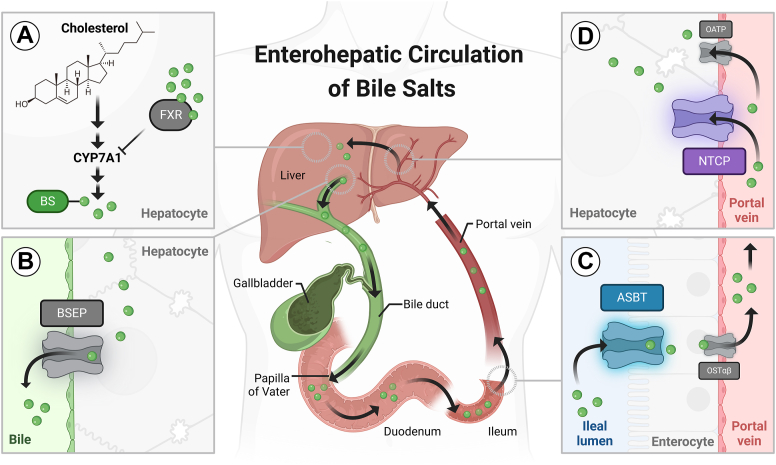
Enterohepatic circulation of BS and important transport proteins involved. A: BS synthesis from cholesterol and negative feedback regulation via FXR and CYP7A1. B: Canalicular BS efflux into bile via BSEP. C: Intestinal absorption of BS via ASBT and OSTα-OSTβ. D: Reuptake of BS into hepatocytes via NTCP and OATPs.

Pharmacological inhibition of ASBT disrupts the enterohepatic circulation of BS and leads to clinical effects at three different levels. (I) Non-absorbed BS and micelles containing BS and dietary lipids stay at higher concentrations in the intestinal lumen. This effect can clinically be used to overcome constipation but can also lead to diarrhea and steatorrhea (11). (II) The reflux of BS via the portal blood flow to the liver is significantly reduced. Under cholestatic conditions, this clinically helps to lower the hepatic BS load and to protect hepatocytes from elevated toxic BS concentrations in different types of cholestatic disorders (12, 13). (III) Finally, de novo BS synthesis is significantly upregulated to compensate for the intestinal BS loss. This process requires quantitative amounts of cholesterol that are delivered to the liver via low-density lipoprotein (LDL). This finally leads to a clinically relevant decline of serum LDL-cholesterol levels (14, 15). Based on these effects, bile acid reabsorption inhibitors (BARIs) acting as pharmacological inhibitors of ASBT have been under preclinical and clinical development for more than 30 years (14). These include propanolamines, barbiturates, quinoline derivatives, lignans, naphthol derivatives, benzothiazepines, benzothiadiazepines, benzothiepines, and some others (12, 13, 14). More recently, the focus for the clinical development of BARIs was on the chemical classes of benzothiazepines (elobixibat and linerixibat), benzothiadiazepines (odevixibat) and benzothiepines (maralixibat) (Fig. 2). BARIs have already been successfully tested or are currently being tested in clinical trials on patients with primary sclerosing cholangitis, primary biliary cholangitis, Alagille syndrome (ALGS), primary familial intrahepatic cholestasis (PFIC), nonalcoholic fatty liver disease, nonalcoholic steatohepatitis, chronic idiopathic constipation, dyslipidemia, cholestatic pruritus, and biliary atresia (12) (www.clinicaltrials.gov). Odevixibat and maralixibat have been approved for the treatment of PFIC and cholestatic pruritus in patients with ALGS, and elobixibat has been approved for the treatment of chronic constipation. All these compounds show low oral bioavailability and are considered to inhibit ASBT from the luminal side of the intestine (12). Consequently, they have a minimal risk for systemic toxicity and drug-drug interactions (DDI). However, the occurrence of diarrhea is a frequently reported adverse side effect in clinical trials with BARIs (16). In addition to these gut-specific ASBT inhibitors, the first systemic ASBT inhibitor, namely compound A3907, has recently been described in preclinical studies (17, 18, 19). Apart from blocking ASBT in the intestine, A3907 also blocks ASBT in the proximal tubules of the kidney. As the renal BS uptake via ASBT under cholestatic conditions has been associated with cholemic nephropathy, systemic ASBT inhibitors lower the BS pool and additionally protect renal proximal tubules from toxic BS concentrations, by increasing the urinary BS excretion (17, 18, 19).

**Fig. 2:**
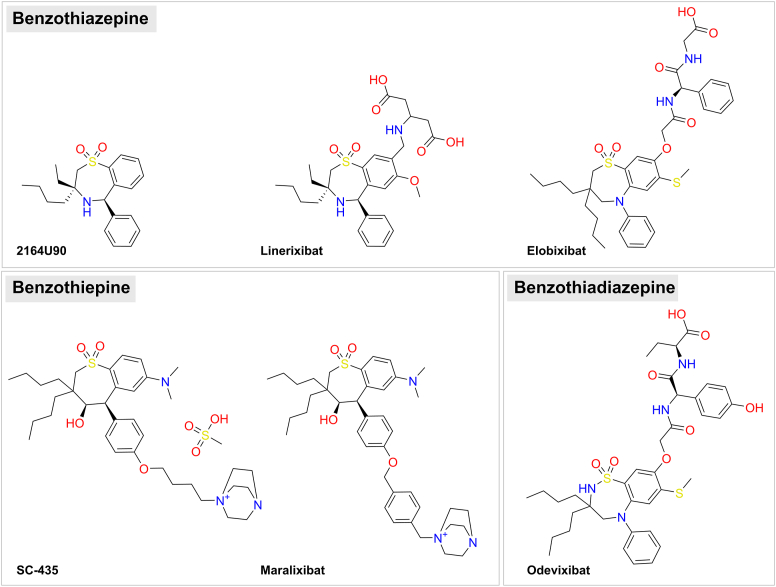
Chemical structures of BARIs and their chemical classification. Linerixibat (alias GSK2330672) currently is in clinical testing in phase II and III studies, elobixibat (alias A3309) has been approved for the treatment of chronic constipation (Goofice), odevixibat (alias A4250) has been approved for the treatment of pruritus in patients with PFIC (Bylvay) or ALGS (Kayfanda), and maralixibat (also known as SHP625, LUM001, or lopixibat) has been approved for the treatment of PFIC and of cholestatic pruritus in patients with ALGS (Livmarli). The compounds 2164U90 (synonym: S0382) and SC-435 have only been used in preclinical studies. The structures of linerixibat, elobixibat, maralixibat, and odevixibat were retrieved from drugbank.com. The structures of 2164U90 and SC-435 were exported from pubchem.ncbi.nlm.nih.gov.

The present study evaluated the BARIs elobixibat, linerixibat, maralixibat, and odevixibat for potential cross-reactivity with the closest homologs of ASBT, namely the hepatic BS carrier NTCP and the steroid sulfate uptake carrier sodium-dependent organic anion transporter (SOAT, gene symbol *SLC10A6*). In addition, given the increasing importance of assessing potential DDI, we also examined the BARI cross-reactivity with three members of the OATP family, namely OATP1B1, OATP1B3, and OATP2B1.

## Materials and methods

### Chemicals

All chemicals, if not otherwise stated, were from Sigma-Aldrich. Odevixibat (MolPort-047-152-914), elobixibat (MolPort-047-155-556), and linerixibat (MolPort-046-033-603) were purchased from MolPort. Maralixibat (HY-16747) and SC-435 (HY-129982) were purchased from Hölzel (Hölzel Diagnostika Handels GmbH). Troglitazone was purchased from Cayman chemicals (97322-87-7).

### Carrier-expressing stably transfected HEK293 cell lines

Human embryonic kidney HEK293 cells, stably transfected with the full open reading frames of human NTCP, ASBT, SOAT, OATP1B1, OATP1B3, OATP2B1, and murine Asbt (mAsbt) were used as reported (18, 20, 21, 22, 23). The NTCP, ASBT, and mAsbt constructs were C-terminally tagged with the FLAG epitope, and the SOAT construct was C-terminally tagged with green fluorescent protein. Non-transfected Flp-In HEK293 cells (Thermo Fisher Scientific) served as the control. All cell lines were maintained at 37°C, 5% CO_2_ and 95% humidity in DMEM/F-12 medium (Thermo Fisher Scientific) supplemented with 10% fetal calf serum (Sigma-Aldrich), 4 mM L-glutamine (PAA) and penicillin/streptomycin (PAA). Expression of the respective carrier protein was induced by tetracycline treatment at 1 μg/ml (Carl Roth GmbH + Co. KG).

### Site-directed mutagenesis

Site-directed mutagenesis of the human NTCP and ASBT constructs used the mutagenesis primers listed in Table 1. Amplification was performed on a peqSTAR XS PEQLAB PCR cycler (Peqlab) with Pfu DNA polymerase (M774A; Promega) in 18 cycles under the following conditions: initial denaturation for 2 min at 95°C, denaturation for 30 s at 95°C, annealing for 1 min at 55°C, extension for 8 min and 30 s at 72°C, final elongation for 10 min at 72°C and final hold at 4°C. After amplification, the products were digested with *Dpn*I (ER1701; Thermo Fisher Scientific) for 1 h at 37°C and transformed into TOP10 chemically competent *E. coli* (Thermo Fisher Scientific). Plasmids were isolated using a GeneJET Plasmid Miniprep Kit (Thermo Fisher Scientific) according to the manufacturer's protocol, and the generated point mutations were verified by DNA sequencing (Seqlab Microsynth).

**Table 1:** Oligonucleotide primers used for site-directed mutagenesis

Mutant Construct	Amino Acid and Nucleotide Substitutions	Primer Sequences (5' → 3') with the Mutated Nucleotides Underlined
NTCP → ASBT	M290S (ATG → AGC)	CCA CTT TTC TTC TTT CCC CTC CTC TAC AGC ATT TTC CAG CTT GGA GAA GGG forward and CCC TTC TCC AAG CTG GAA AAT GCT GTA GAG GAG GGG AAA GAA GAA AAG TGG reverse
NTCP → mAsbt	M290T (ATG → ACG) and I291V (ATT → GTT)	CCA CTT TTC TTC TTT CCC CTC CTC TAC ACG GTT TTC CAG CTT GGA GAA GGG foward and CCC TTC TCC AAG CTG GAA AAC CGT GTA GAG GAG GGG AAA GAA GAA AAG TGG reverse
ASBT → mAsbt	S294T (AGC → ACC) and I295V (ATT → GTT)	CCG CTC ATC TAC ACC GTT TTC CAG CTC GCC TTT G forward and CAA AGG CGA GCT GGA AAA CGG TGT AGA TGA GCG G reverse
ASBT → NTCP	S294M (AGC → ATG)	CAC CTT CCC GCT CAT CTA CAT GAT TTT CCA GCT CGC CTT TG forward and CAA AGG CGA GCT GGA AAA TCA TGT AGA TGA GCG GGA AGG TG reverse

### Transport and inhibition assays

Transport and binding experiments in the respective carrier-expressing HEK293 cells were done with the following probe substrates: 1 μM [^3^H]taurocholic acid ([^3^H]TC, 20 Ci/mmol, American Radiolabeled Chemicals (ARC) via Biotrend Chemikalien GmbH) for NTCP, ASBT, and mAsbt; 1 μM [^3^H]dehydroepiandrosterone sulfate ([^3^H]DHEAS, 88.3 Ci/mmol, Revvity) for SOAT and NTCP; 1 μM [^3^H]rosuvastatin (25 Ci/mmol, ARC) and 5 nM [^3^H]myr-preS1_2-48_ peptide ([^3^H]preS1, 129 Ci/mmol, RC Tritec AG) for NTCP; 0.2 μM [^3^H]estrone-3-sulfate ([^3^H]E_1_S, 50 Ci/mmol, ARC) for OATP1B1 and OATP2B1; and 1 μM [^3^H]bromosulfophthalein ([^3^H]BSP, 10.2 Ci/mmol, Hartmann Analytic GmbH) for OATP1B3. Cells were seeded onto poly-L-lysine-coated 96-well plates, induced with 1 μg/ml tetracycline, and grown to confluence over 72 h at 37°C. Then, cells were washed with tempered (37°C) phosphate-buffered saline (PBS, containing 137.0 mM NaCl, 2.7 mM KCl, 1.5 mM KH_2_PO_4_, and 7.3 mM Na_2_HPO_4_, at pH 7.4) and preincubated with 80 μl sodium transport buffer (STB, containing 142.9 mM NaCl, 4.7 mM KCl, 1.2 mM MgSO_4_, 1.2 mM KH_2_PO_4_, 1.8 mM CaCl_2_, and 20.0 mM HEPES, at pH 7.4) for 5 min at 37°C. For inhibition studies, the medium was replaced by 80 μl STB containing the respective inhibitor or solvent alone and cells were further incubated for 5 min at 37°C. Transport experiments were started by adding 20 μl of a 5-fold concentrated substrate solution (solved in STB). For transport experiments under sodium-free conditions the same buffer composition was used as for STB, but sodium chloride was substituted with equimolar concentrations of choline chloride as previously reported (20, 24). Transport experiments were stopped by washing twice with ice-cold PBS and the plates were kept cool on ice until adding the lysis buffer [1% sodium dodecyl sulfate (SDS) and 1 N NaOH]. Then, the cell-associated radioactivity of the respective tritium-labeled substrate or ligand was quantified by liquid scintillation counting using MicroScint-20 scintillation cocktail (Revvity) in a Packard Microplate Scintillation Counter TopCount NXT (Packard Instrument Company). For IC_50_ transport inhibition measurements, the mean uptake values measured in non-carrier-expressing HEK293 control cells were defined as 0% uptake control and were subtracted from all other values. Values from cells without inhibitor and solvent alone were set to 100%. Finally, all net transport data were expressed as uptake or binding as % of control. All transport or inhibition graphs were generated with GraphPad Prism 6 (GraphPad). Determination of half maximal inhibitory concentrations (IC_50_) was done by nonlinear regression analysis using the equation log (inhibitor) versus response—variable slope settings. All data points of the IC_50_ curves represent means ± SD of quadruplicate determinations. For K_m_ measurements, BS uptake in the absence of sodium was regarded as carrier-independent unspecific uptake and was subtracted from the transport rates in the presence of sodium to obtain carrier-specific uptake rates (indicated as dashed lines). From the specific uptake rates, Michaelis Menten K_m_ values were calculated by non-linear regression analysis.

### Transport inhibition and recovery assays

HEK293 cells stably expressing ASBT were seeded onto poly-L-lysine-coated 96-well plates, induced with 1 μg/ml tetracycline, and grown to confluence over 72 h at 37°C. Non-carrier-expressing HEK293 cells were used as control. Then, cells were washed with tempered (37°C) PBS and equilibrated for 5 min with STB. Afterwards the respective BARI transport inhibitors were incubated for 5 min at 3 μM, where no residual transport activity was expected based on the IC_50_ measurements. After inhibitor preincubation, cells were washed with tempered (37°C) PBS and further incubated in inhibitor-free DMEM (Thermo Fisher Scientific) for 30, 60, 120, or 240 min at 37°C. Transport experiments with 1 μM [^3^H]TC were either performed after this washout and recovery phase or directly after inhibitor preincubation, either in the presence or in the absence of inhibitor. Finally, cell associated radioactivity was determined using the MicroBeta2 liquid scintillation counter (Revvity). For each inhibitor, the mean values from noncarrier-expressing HEK293 cells were set to 0% and transport measurements in the absence of inhibitor (solvent control) were set to 100%. Results are shown as % [^3^H]TC uptake inhibition as mean ± SD of quadruplicate determinations of one out of two representative experiments.

### Western blot

For Western blotting, the respective carrier-expressing HEK293 cells were seeded in 6-well plates and protein expression was induced using 1 μg/ml tetracycline. After 72 h, the cells were harvested for protein extraction with the ProteoExtract Kit (444810, Sigma-Aldrich), and the lysate membrane fraction was collected. Protein lysates were quantified using the BCA protein assay kit (71285-3, Sigma-Aldrich) and digested with 1 U PNGaseF (P07045, New England Biolabs) for 1 h at 37°C. Subsequently, samples were normalized to 17 μg protein, mixed with 4X-Laemmli buffer (containing 8% SDS, 40% glycerin, 10% β-mercaptoethanol, 0.008% bromophenol blue, and 250.0 mM Tris) and incubated for 10 min at room temperature. Afterwards, the samples were separated by SDS polyacrylamide gel electrophoresis (SDS-PAGE) on a 12% acrylamide separation gel with 3% collection gel. The separated protein samples were then blotted onto a Roti-PVDF membrane (Carl Roth GmbH + Co. KG) at 400 mA for 45 min. Subsequently, the membrane was blocked for 1 h with 10% milk powder in TBS-T (Tris-Buffered Saline with Tween-20, containing 137.0 mM NaCl, 10.0 mM Tris, and 0.05% Tween-20, at pH 8.0). Primary antibody incubation was performed at 4°C overnight with rabbit anti-Flag antibody (1:3000, #F7425, Sigma-Aldrich). After 16 h, the membrane was washed 3–4 times with TBS-T for 15 min. Secondary antibody incubation with the anti-rabbit antibody (1:5000, #31460, Thermo Fisher Scientific) was performed for 1 h at room temperature. The membrane was washed again, and the chemiluminescence of the HRP-coupled secondary antibody was detected using SuperSignal West Pico Plus Chemiluminescent Substrate solution (#34577, ThermoFisher Scientific) and the ChemiDoc Imaging system (Bio-Rad). Subsequently, the same PVDF membrane was stained with Coomassie-blue solution (0.25% Coomassie R-250, 25% ethanol, 10% acetic acid in double-distilled water) for 10 min. Afterwards the membrane was incubated for 15 min and washed three times in destaining solution (30% ethanol, 10% acetic acid in double-distilled water). The bands of interest were then detected with the ChemiDoc Imaging system (Bio-Rad).

### Docking of linerixibat to human ASBT and visualization

The 2D structure of linerixibat was exported from PubChem as SDfile (25). Protein structures were sourced either directly from the Protein Data Bank (PDB) (26, 27), or represent Swiss-Model or YASARA (28, 29) homology models based on the cryo-EM NTCP structures with the PDB IDs 7ZYI, 7VAD (26, 27), or 8RQF (30). Using the MAESTRO molecular modeling interface (Version 13.2) of SCHRÖDINGER (Schrödinger Release 2022-2: Maestro, Schrödinger), linerixibat was prepared as the ligand using LigPrep (Schrödinger Release 2022-2: LigPrep, Schrödinger) with the following settings: force field OPLS_2005 (default setting); ionization state at target pH 7.4 ± 1 (Epik; custom setting); desalt (default setting); generate tautomers (default setting); and retain specified chiralities (default setting). The protein structures were prepared for docking using the protein preparation workflow at pH 7.4, including hydrogen bond assignment and minimization with default settings. Additional ligand information from a previously published ASBT pharmacophore model was considered to assess the correct binding to ASBT (31). With the ASBT amino acid residues 294 and 295 as the docking grid basis, a receptor grid was defined (outer box 30 × 30 × 30 Å^3^; inner box 10 × 10 × 10 Å^3^) and docking was carried out using Glide at the XP-mode (32, 33). Binding modes were assessed manually and potential rotamers of residues were subsequently evaluated.

## Results

### SLC10 carrier uptake and inhibition studies

**Fig. 3:**
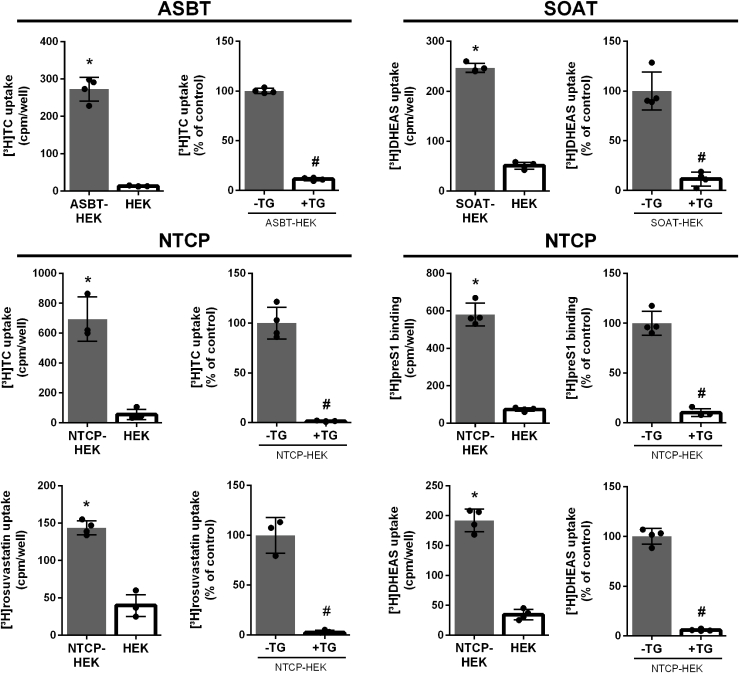
Transport, binding and inhibition experiments with the SLC10 carriers ASBT, NTCP, and SOAT. Transport and binding experiments were performed with the indicated radiolabeled compounds (1 μM [^3^H]TC, 1 μM [^3^H]DHEAS, 5 nM [^3^H]preS, and 1 μM [^3^H]rosuvastatin) in HEK293 cells stably transfected with ASBT, SOAT, and NTCP, respectively. All transport and binding experiments were performed over 10 min. As indicated, non-carrier-expressing HEK293 cells (HEK) were used as control, or the pan-SLC10 inhibitor troglitazone was used at 100 μM (+TG). Bars represent means ± SD of quadruplicate determinations. ∗Significant higher carrier-dependent transport or binding compared to HEK cells and ^#^significant inhibition in the presence of inhibitor (+TG) with *P* < 0.05 based on two-tailed unpaired *t* test.

The objective of the present study was to assess target-specificity of the BARIs elobixibat, linerixibat, odevixibat, maralixibat, and SC-435. While elobixibat (GOOFICE), odevixibat (BYLVAY, KAYFANDA), and maralixibat (LIVMARLI) are already in clinical use, linerixibat is still under clinical development (34). The preclinical BARI drug SC-435 was also included for comparison but the BARIs 2164U90 (synonym: S0382) and volixibat (alias SHP626) were not commercially available and, therefore, could not be included in the present study. All these compounds are highly potent inhibitors of ASBT, and the first aim was to analyze their potential cross-reactivity with the closely related carriers NTCP and SOAT. For these studies well-established stably transfected HEK293 cell lines, namely ASBT-HEK, NTCP-HEK, and SOAT-HEK, were applied (20, 22). As probe substrates, the following radiolabeled compounds were used: [^3^H]TC for ASBT and NTCP, [^3^H]DHEAS for NTCP and SOAT, as well as the cholesterol-lowering drug [^3^H]rosuvastatin for NTCP. In addition to the physiological BS transport and drug transport functions of NTCP, binding of the viral [^3^H]preS1 peptide to NTCP was used as a surrogate parameter for hepatitis B virus (HBV) binding to NTCP, as reported before (35) to address all aspects of NTCP functions. As shown in Figure 3, all probe substrates showed significantly higher transport rates in the carrier-expressing HEK293 cells compared to non-carrier-expressing control cells (HEK). The [^3^H]preS1 peptide showed significantly higher binding to the NTCP-HEK cells compared to the control. The carrier-dependent transport and binding were significantly inhibited with the pan-SLC inhibitor and thiazolidinedione drug troglitazone at 100 μM (+TG). This data indicates that the cell lines used are appropriate tools to comparatively analyze inhibitors for the carriers ASBT, NTCP, and SOAT.

### Cross-reactivity of BARIs with NTCP and SOAT

**Fig. 4:**
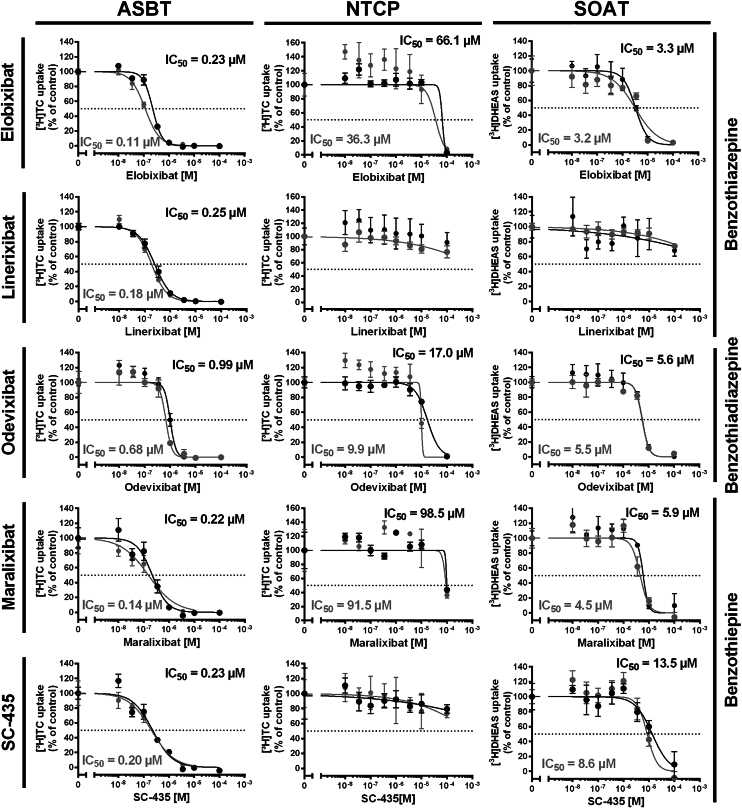
Inhibition of the substrate transport via ASBT, NTCP, and SOAT with the BARIs elobixibat, linerixibat, odevixibat, maralixibat, and SC-435. Transport experiments were performed in HEK293 cells stably transfected with ASBT, NTCP, or SOAT. Transport experiments were performed for 10 min with 1 μM [^3^H]TC for ASBT and NTCP, or with 1 μM [^3^H]DHEAS for SOAT at increasing concentrations of the indicated BARIs. The two IC_50_ curves and values per diagram represent two independent experiments each with quadruplicate determinations. IC_50_ values were determined by non-linear regression analysis and could only be reliably calculated when the inhibition at the highest inhibitor concentration was at ≥ 50%. The x-axes of the IC_50_ diagrams are arranged in logarithmic representation.

To analyze if the BARIs elobixibat, linerixibat, odevixibat, maralixibat, and SC-435 are target-specific for ASBT or behave like troglitazone as pan-SLC10 inhibitor, comprehensive transport studies were performed with ASBT-HEK, NTCP-HEK, and SOAT-HEK cells in the presence of increasing concentrations of the respective BARIs. In Figure 4, each diagram shows IC_50_ curves and IC_50_ values of two independent experiments. These experiments confirmed the highly potent inhibition of ASBT for all BARIs analyzed, with well comparable IC_50_ values of 0.11 and 0.23 μM for elobixibat, 0.18 and 0.25 μM for linerixibat, 0.68 and 0.99 μM for odevixibat, 0.14 and 0.22 μM for maralixibat, and 0.20 and 0.23 μM for SC-435. Interestingly, most of these compounds also showed potent inhibition of the closely related carrier SOAT, namely elobixibat (IC_50_ of 3.2 and 3.3 μM), odevixibat (IC_50_ of 5.5 and 5.6 μM), maralixibat (IC_50_ of 4.5 and 5.9 μM), and SC-435 (IC_50_ of 8.6 and 13.5 μM). As the only exception, linerixibat failed to significantly inhibit SOAT up to 100 μM. Regarding cross-reactivity with the hepatic BS carrier NTCP, elobixibat, odevixibat, and maralixibat showed some weak inhibition at very high inhibitor concentrations of 100 μM with calculated IC_50_ values of 36.3 and 66.1 μM for elobixibat, 9.9 and 17.0 μM for odevixibat, as well as 91.5 and 98.5 μM for maralixibat. However, linerixibat and SC-435 exhibited no significant NTCP inhibition up to an inhibitor concentration of 100 μM.

**Fig. 5:**
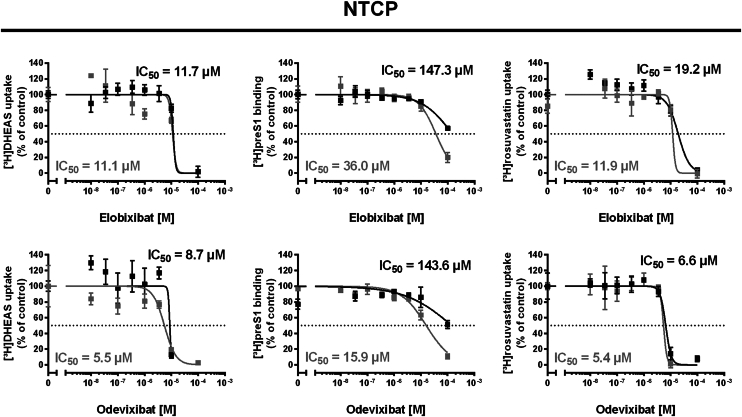
Inhibition of the transporter and virus receptor functions of NTCP with the active BARIs elobixibat and odevixibat. Transport experiments were performed for 10 min with 1 μM [^3^H]rosuvastatin, or 1 μM [^3^H]DHEAS, and HBV preS1-peptide binding experiments were performed with 5 nM [^3^H]preS1. The active BARIs elobixibat and odevixibat (see Fig. 4) were used as inhibitors at increasing concentrations. The two IC_50_ curves and values per diagram represent two independent experiments each with quadruplicate determinations. IC_50_ values were calculated by non-linear regression. The x-axes of the IC_50_ diagrams are arranged in logarithmic representation.

As it is not finally known if and to what extent the proposed multiple substrate and inhibitor binding sites of NTCP overlap with each other, inhibition of NTCP by elobixibat and odevixibat was additionally analyzed using [^3^H]DHEAS and [^3^H]rosuvastatin as the transport substrates as well as [^3^H]preS1 as the binding ligand. As shown in Figure 5, odevixibat was a slightly more potent inhibitor of NTCP than elobixibat, when [^3^H]DHEAS and [^3^H]rosuvastatin were used as the transport substrates. Notably, both compounds not only inhibited the transporter function of NTCP but also blocked the binding of the viral [^3^H]preS1 peptide to NTCP, however, at quite high inhibitor concentrations and with large inter-assay variability. Nevertheless, this suggests a potential anti-HBV activity at high inhibitor concentrations.

### Cross-reactivity of BARIs with OATP carriers

**Fig. 6:**
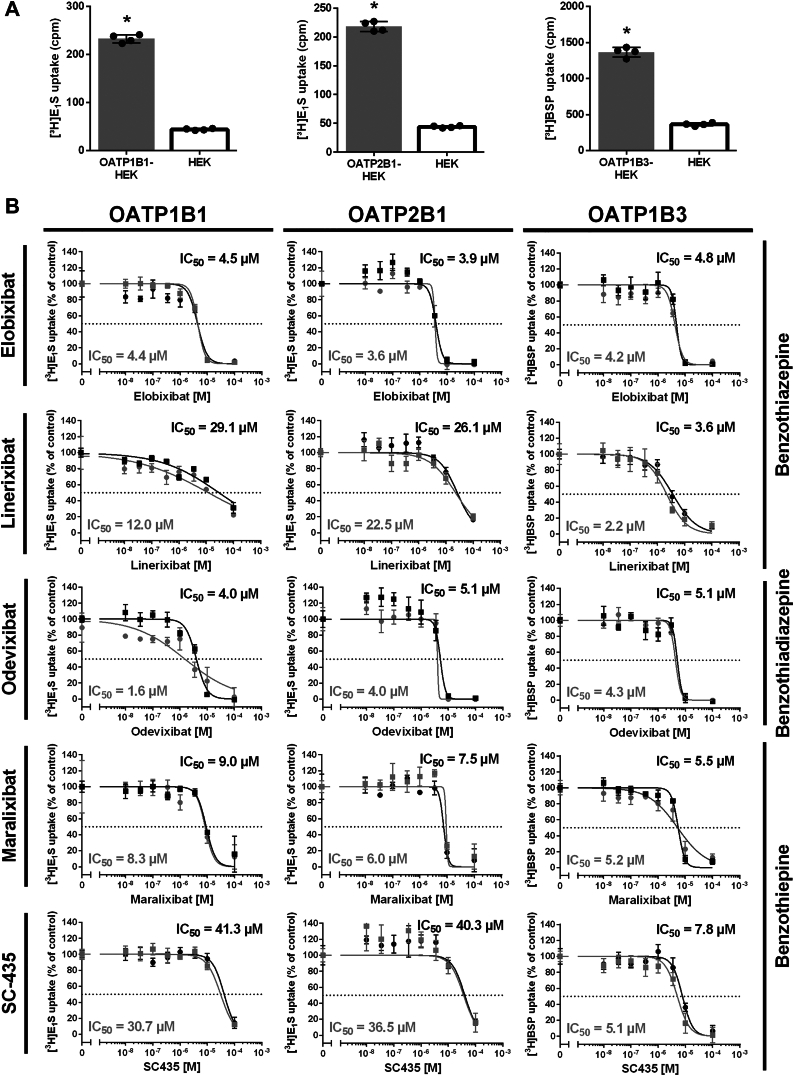
Inhibition of the substrate transport via OATP1B1, OATP2B1, and OATP1B3 with the BARIs elobixibat, linerixibat, odevixibat, maralixibat, and SC-435. A: transport experiments were performed for 10 min with the indicated radiolabeled compounds [^3^H]E1S at 200 nM and [^3^H]BSP at 1 μM, respectively, in HEK293 cells stably transfected with OATP1B1 (OATP1B1-HEK), OATP2B1 (OATP2B1-HEK), or OATP1B3 (OATP1B3-HEK). Non-carrier-expressing HEK293 cells (HEK) were used as control (open bars). Bars represent means ± SD of quadruplicate determinations. ∗Significantly higher carrier-dependent transport with *P* < 0.05 based on two-tailed unpaired *t* test. B: transport experiments were performed for 10 min with the same substrate concentrations for all three carriers at increasing concentrations of the indicated BARIs. The two IC_50_ curves and values per diagram represent two independent experiments each with quadruplicate determinations. IC_50_ values were calculated by non-linear regression. The x-axes of the IC_50_ diagrams are arranged in logarithmic representation.

Apart from NTCP, three members of the OATP family, namely OATP1B1, OATP1B3, and OATP2B1, are expressed in the liver and show overlapping transport activities with NTCP (36). To analyze if these carriers also share BARIs as transport inhibitors, additional transport and inhibition experiments were performed in HEK293 cell lines stably expressing the respective OATP carriers, namely OATP1B1-HEK, OATP2B1-HEK, and OATP1B3-HEK (21, 23). As shown in Figure 6A, OATP1B1, OATP2B1, and OATP1B3 showed significant transport activities for the probe substrates [^3^H]E_1_S and [^3^H]BSP, respectively. Then, elobixibat, linerixibat, odevixibat, maralixibat, and SC-435 were used as potential OATP inhibitors at increasing concentrations. As shown in Figure 6B, all BARIs analyzed in the present study showed significant cross-reactivity against OATP1B1, OATP2B1, and OATP1B3. Elobixibat, odevixibat, and maralixibat were the most potent OATP inhibitors with IC_50_ values ranging from 2-9 μM. Linerixibat and SC-435 showed IC_50_ values > 10 μM for OATP1B1 and OATP2B1, while both compounds inhibited OATP1B3 within the range of the other compounds (with IC_50_ values of 2-8 μM). This data clearly indicates that BARIs, at least at high concentrations, are inhibitors of SLC10 and OATP carriers.

### BARI—different inhibition pattern at ASBT

**Fig. 7:**
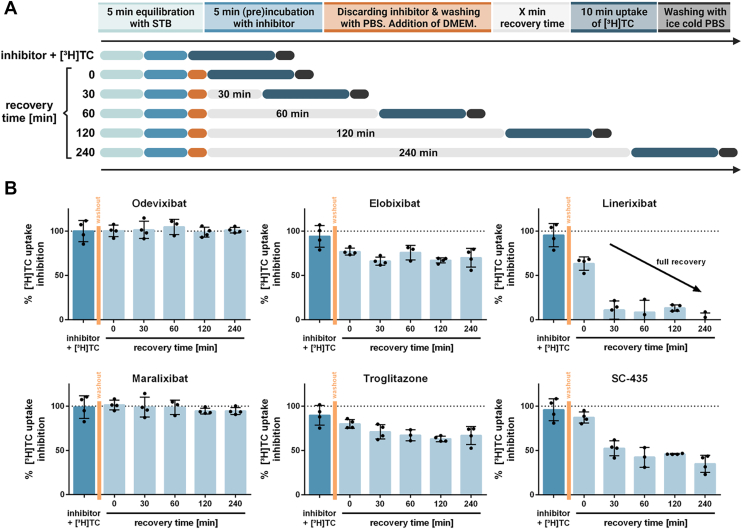
Time-dependent recovery of the [^3^H]TC uptake inhibition after inhibitor washout. A: Timeline of the transport inhibition and recovery assay. B: HEK293 cells stably expressing ASBT and non-carrier-expressing HEK293 cells were preincubated for 5 min with 3.0 μM of the indicated BARI, with solvent alone (0.5% DMSO), or with the control inhibitor troglitazone at 100 μM. Then, the uptake of 1 μM [^3^H]TC was measured in the presence of the inhibitor (inhibitor + [^3^H]TC), revealing maximum transport inhibition. The uptake of 1 μM [^3^H]TC was also measured after washout of the inhibitor and inhibitor-free incubation over 0–240 min (recovery time), and the remaining degree of inhibition is indicated in the diagrams. For the calculation of the [^3^H]TC uptake inhibition data, the solvent control was set to 100% (no inhibition control) and the uptake in non-carrier-expressing HEK293 cells was set to 0% (no transport control). Data represent means ± SD of quadruplicate determinations.

Even if the ASBT inhibition potency was quite similar for the BARIs elobixibat, linerixibat, odevixibat, maralixibat, and SC-435, we aimed to further analyze this transporter inhibition with a set of additional experiments that are shown in Figure 7. First, the inhibitor was pre-incubated for 5 min, then [^3^H]TC was added, and the uptake phase lasted over 10 min in the presence of substrate plus inhibitor. This experimental setup is marked in Figure 7A as “inhibitor + [^3^H]TC” and represents the classical inhibition assay that was also used to determine the IC_50_ values for transporter inhibition (see Fig. 4). In the second setup, the inhibitor was thoroughly washed out after the preincubation phase and cells were placed on inhibitor-free medium for different time intervals of 0–240 min (indicated by “recovery time” in Fig. 7A). After this recovery time, the uptake of [^3^H]TC was measured over 10 min in the absence of inhibitor. In both setups, experiments were performed at 3 μM inhibitor concentration, representing full ASBT transport inhibition for all BARIs.

As shown in Figure 7B, the BARIs analyzed in the present study showed quite different inhibition patterns. Odevixibat and maralixibat retained full ASBT uptake inhibition after only 5 min inhibitor preincubation, thorough inhibitor washout and up to 240 min incubation in inhibitor-free medium. In clear contrast, linerixibat only showed full ASBT inhibition when the inhibitor was present during the 10 min transport phase together with [^3^H]TC. Inhibitor washout directly recovered about 37% of the ASBT transport activity, and an additional 30 min incubation in inhibitor-free medium mostly recovered ASBT. Elobixibat and SC-435 revealed an intermediate inhibition pattern. This data clearly points to a completely different inhibition pattern with (I) long-lasting full ASBT inhibition even in the absence of inhibitor in the medium (odevixibat and maralixibat), (II) full ASBT inhibition with quick and full recovery in the absence of inhibitor in the medium (linerixibat), and (III) an intermediate inhibition pattern with partial recovery in the absence of inhibitor in the medium (elobixibat). For comparison, the ASBT inhibitor troglitazone was used in the same experimental setup and revealed nearly complete ASBT inhibition at 100 μM with only partial recovery after washout and 240 min incubation in the inhibitor-free medium, like the pattern obtained with elobixibat.

### Localization of the linerixibat inhibitor binding site at ASBT

Apart from the inhibition pattern and mode of inhibition, it was of interest to localize parts of the inhibitor binding pocket for the BARIs at ASBT. Based on similar inhibition potencies a common inhibitor binding pocket can be proposed for all BARIs tested in the present study. However, the time-resolved inhibition studies with inhibitor washout more point to separate inhibitor binding or distinct interaction sites. In the present study, we aimed to identify at least parts of the inhibitor binding sites of the BARIs at ASBT by means of in silico molecular docking and in vitro transport studies on mutant carrier proteins.

**Fig. 8:**
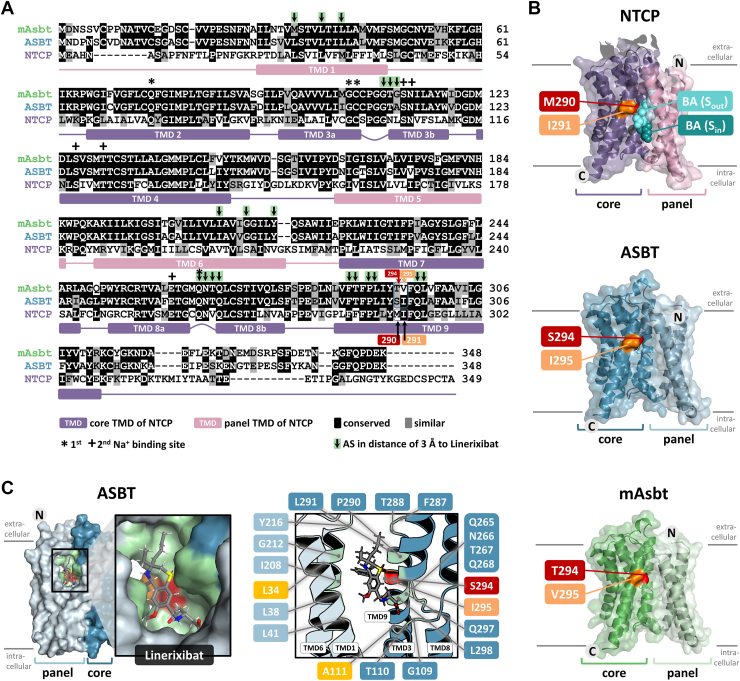
Potential drug binding site of linerixibat at human ASBT. A: Protein sequence alignment for mouse Asbt (mAsbt), human ASBT, and human NTCP. The alignment compares and scores the protein sequences against the consensus sequence of all aligned sequences (threshold value = 0.5). Black shading represents conserved amino acids between at least two of the three carriers, grey shading indicates amino acids with similar properties in terms of charge, size and hydrophobicity. White areas indicate deviations of the protein sequence from the consensus sequence. Localization of the transmembrane domains (TMD) of NTCP is visualized by rectangles (violet = core; pink = panel domain) and amino acid residues forming the sodium binding sites I and II of NTCP are indicated with the symbols “∗” and “+”, respectively. Arrows indicate amino acids that are located within a 3 Å distance from linerixibat docket into the homology model of ASBT (see C). Amino acid positions 294/295 for mAsbt/ASBT and positions 290/291 for NTCP are highlighted with red and orange labels, respectively. These positions served as mutation sites for the ASBT and NTCP proteins in the present study. B: 3D protein structure for NTCP (PDB 7ZYI) and Swiss model predicted protein structures for ASBT and mAsbt (both based on the PDB 7ZYI structure of NTCP). The core domains are shown in dark colors and the panel domains in light colors. The N-terminus is extracellular, and the C-terminus is intracellular. The mutation sites for NTCP and ASBT are shown in a surface style, highlighted in red (position 290/294) or orange (position 291/295). For NTCP, the two proposed bile acid (BA) binding pockets (S_out_ and S_in_) are highlighted (26, 27). C: Docking of linerixibat to the predicted Swiss model protein structure of ASBT (based on the PDB 8RQF structure of NTCP). Green-colored surface and helix structures indicate amino acids laying within a distance of ≤ 3 Å from the docked linerixibat molecule. These positions are additionally numbered in the cartoon structure. All amino acid positions that are common between ASBT and mAsbt are colored blue and positions different between ASBT and mAsbt are shown in yellow, orange, and red.

Unfortunately, the structural information for human ASBT is limited to (I) homology models based on bacterial ASBT proteins that are, however, very distant and have an extra N-terminal transmembrane domain (TMD) that is not present in human ASBT (37, 38), (II) AlphaFold models that might not be appropriately resolved for molecular docking studies (39), and (III) homology models based on recent cryo-EM structures of the functional homologous carrier NTCP in substrate-bound and apo conformations (26, 27, 40, 41). Considering these limitations, we tried different approaches of molecular docking to the different classes of structures (I-III) and used previous functional data to validate the respective models. This functional data was generated by Hallén *et al.* (2002) for a BARI of the benzothiazepine class, named 2164U90 (synonym: S0382, Fig. 2) (42). This compound was used as an inhibitor of ASBT and mAsbt and revealed quite different *K*_*i*_ values of 10 μM versus 0.068 μM, respectively. With a series of chimeric and single mutant ASBT proteins, amino acids S294 and I295 of human ASBT (T294 and V295 in mAsbt) were found to be responsible for this species difference and were proposed to be part of the inhibitor binding site of compound 2164U90. Of note, this compound builds the backbone structure of linerixibat that was used in the present study. Based on this, we focused our molecular docking on linerixibat as the docking drug. Based on the mentioned functional data, all docking poses of linerixibat at ASBT were validated for their interaction with the S294/I295 potential drug binding site of ASBT and for the similarity to the docking pose of 2164U90. As shown in Figure 8A, apart from the mentioned differences between ASBT (S294/I295) and mAsbt (T294/V295) in the middle of TMD 9, there are only few other species differences (ASBT vs. mAsbt) that are localized at relevant sites for potential interference with BS substrate binding. Of note, human NTCP is much more distant to the ASBT and mAsbt sequences and has the amino acid residues M290 and I291 at the corresponding positions. As shown in Figure 8B, this potential drug binding site is near the outer substrate binding site (S_out_) that has been identified by cryo-electron microscopy (cryo-EM) for substrate-bound NTCP with PDB 7ZYI (26). Translated to the homology models of ASBT and mAsbt this might explain why BARIs binding to this site would inhibit BS transport. Based on this, the positions S294/I295 of ASBT were chosen as centroid of the docking grid. Docking of linerixibat to ASBT, structurally based on the outward open NTCP structure with PDB 8RQF, then revealed a reasonable docking pose that is near the S294/I295 potential inhibitor binding site (Fig. 8C). Notably, nearly all other amino acids surrounding this proposed binding pocket of linerixibat at ASBT are conserved among ASBT and mAsbt, with the only exception of L34 in TMD 1 and A111 in TMD 3b. We additionally validated this docking pose based on the 3D QSAR pharmacophore model that had been described for ASBT inhibitors in the group of Werner Kramer already in 1999 (31). This pharmacophore model is characterized by five chemical features, namely (I) one hydrogen bond donor, (II) one hydrogen bond acceptor, and (III) three hydrophobic features. 2164U90, and probably also linerixibat, fulfill these pharmacophore conditions (I) by the sulfonyl group as hydrogen bond acceptor, (II) by the secondary amino group of the benzothiazepine ring as the hydrogen bond donor, and (III) by three hydrophobic interactions via the phenyl ring, the ethyl group, and the butyl group. Of note, all these groups are identical between 2164U90 and linerixibat. Accordingly, potential binding interactions might occur with ASBT via T267 to the sulfonyl group, via Y216 to the secondary amine, as well as via I295 to the phenyl group, via L34 to the ethyl group and via L291 to the butyl group of linerixibat.

**Fig. 9:**
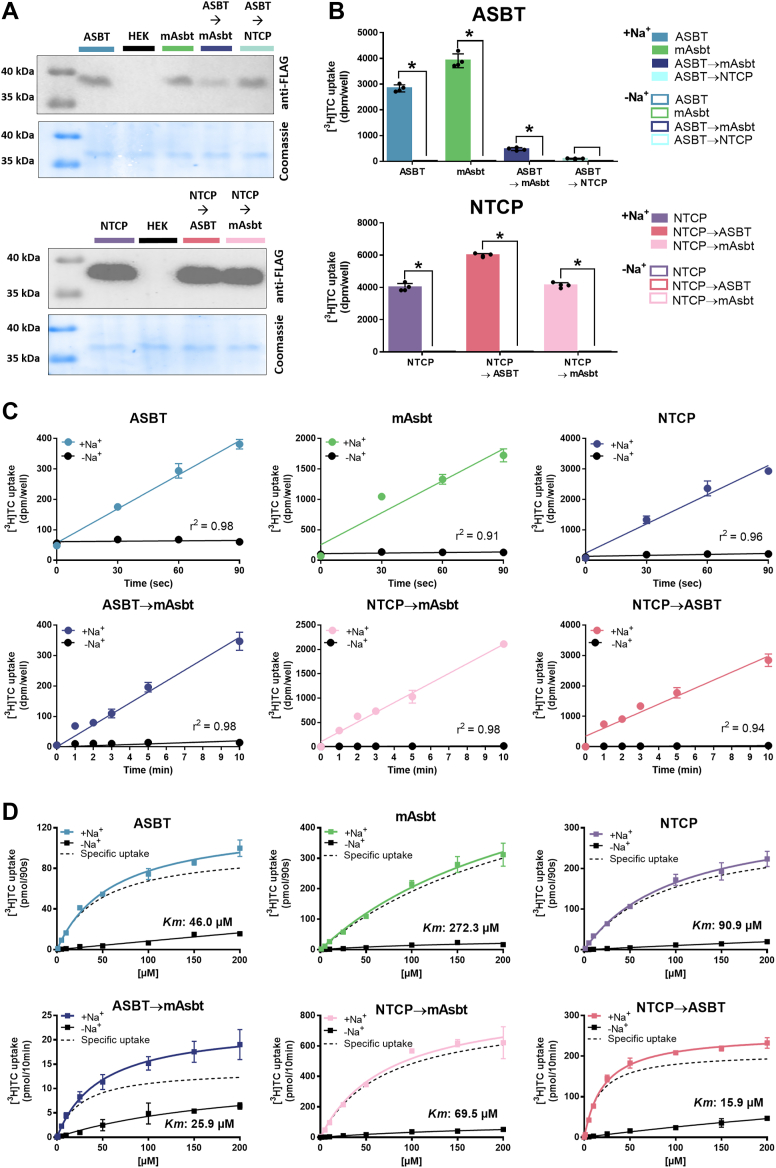
Characterization of the ASBT and NTCP mutant carriers for validation of the linerixibat drug binding sites. A: The following mutants were generated by site-directed mutagenesis and stably transfected into HEK293 cells: NTCP M290S (referred to as NTCP→ASBT), NTCP M290T/I291V (referred to as NTCP→mAsbt), ASBT S294T/I295V (referred to as ASBT→mAsbt), and ASBT S294M (referred to as ASBT→NTCP) (see Table 1). All mutant constructs were FLAG-tagged as their respective wild-type carriers. All cell lines were subjected to membrane protein extraction and deglycosylation prior to Western blot detection with an anti-FLAG antibody. For loading control, all membranes were subsequently stained with Coomassie blue. B: Transport experiments were performed for 30 min for all cell lines stably expressing the respective mutant constructs with 1 μM [^3^H]TC in the presence (+Na^+^) or absence (-Na^+^) of sodium in the transport buffer. ∗Significant sodium-dependent TC transport based on Student’s *t* test with *P* < 0.05. C: Time-dependent transport studies with HEK293 cells stably expressing the wild-type and the respective NTCP and ASBT mutant constructs with 1 μM [^3^H]TC. TC transport was analyzed in the presence (+Na^+^) or absence (-Na^+^) of sodium in the transport buffer over the indicated time periods. For the ASBT→NTCP mutant, time-dependent transport was not analyzed due to the overall low transport rates of this mutant. The initial transport rates were linear over 90 s for the wild-type carriers and over 10 min for the mutants, indicated by linear regression analysis with *r*^2^ of ≥ 0.9. D: Transport kinetic studies with increasing concentrations of [^3^H]TC in HEK293 cell lines expressing the respective wild-type or mutant ASBT, NTCP, and mAsbt constructs were performed at the respective initial linear uptake phase. For the ASBT→NTCP mutant, transport kinetic studies could not be performed due to the overall low transport rates of this mutant. Transport experiments were performed with increasing concentrations of [^3^H]TC in the presence (+Na+) or absence (-Na+) of sodium in the transport buffer. TC uptake in the absence of sodium was regarded as carrier-independent, unspecific uptake and was subtracted from the transport rates in the presence of sodium to obtain carrier-specific uptake rates (indicated as dashed lines). From the specific uptake rates, Michaelis-Menten *K*_*m*_ values were calculated by non-linear regression analysis and the respective *K*_*m*_ values are indicated in the diagrams. All transport data of panels B-D represent means ± SD of quadruplicate determination of one experiment.

To experimentally validate linerixibat binding to the S294/I295 centered binding site at ASBT, the following mutant proteins were generated and functionally characterized. ASBT S294T/I295V double mutation was intended to mimic mAsbt at this position (ASBT→mAsbt) and to test the hypothesis of more potent inhibitor binding. In contrast, the mutant ASBT S294M was generated to mimic NTCP at this position (ASBT→NTCP) and to test the hypothesis of absent inhibition based on the functional inhibition data for linerixibat at NTCP (Fig. 4). Furthermore, the corresponding amino acids of ASBT or mAsbt were introduced into the NTCP sequence to test if the corresponding mutants NTCP M290S (NTCP→ASBT) and NTCP M290T/I291V (NTCP→mAsbt) might get sensitive for linerixibat inhibition. Furthermore, we accordingly generated the following mutants at the corresponding positions as controls: ASBT→SOAT (S294G/I295L), SOAT→ASBT (G294S/L295I) and SOAT→mAsbt (G294T/L295V). However, as these three mutants could not be properly expressed in HEK293 cells or showed no significant transport activity in the pre-screening studies (data not shown), they were not further considered in the present study. The other mutant constructs demonstrated intact membrane protein expression, as shown by Western blotting (Fig. 9A), and transport experiments with [^3^H]TC revealed intact transport activities (Fig. 9B). The sole exception was the ASBT→NTCP mutant, which showed no significant transport activity and was therefore excluded from all further function transport analyses. Notably, the ASBT→mAsbt mutant exhibited somewhat lower protein expression and lower transport rates compared to the ASBT and mAsbt wild-type carriers. Time-dependent transport studies revealed linear initial uptake rates over 90 s for the wild-type carriers ASBT, mAsbt, and NTCP, as well as over 10 min for all mutant carriers (Fig. 9C). Transport kinetic studies confirmed similar substrate affinities for wild-type ASBT (*K*_*m*_ = 46.0 μM) and the ASBT→mAsbt mutant (*K*_*m*_ = 25.9 μM) as well as for wild-type NTCP (*K*_*m*_ = 90.9 μM) and the NTCP→mAsbt mutant (*K*_*m*_ = 69.5 μM). In contrast, mAsbt showed much lower affinity transport of [^3^H]TC with *K*_*m*_ = 272.3 μM and the NTCP→ASBT mutant revealed high-affinity transport with *K*_*m*_ = 15.9 μM (Fig. 9D). Based on this, despite the differences in the absolute transport rates for [^3^H]TC by the wild-type and the mutant constructs, all cell clones are appropriate for comparative inhibition studies with BARIs to evaluate the role of the amino acids S294 and I295 of ASBT for drug binding.

### Characterization of the ASBT 294/295 drug binding site for linerixibat

**Fig. 10:**
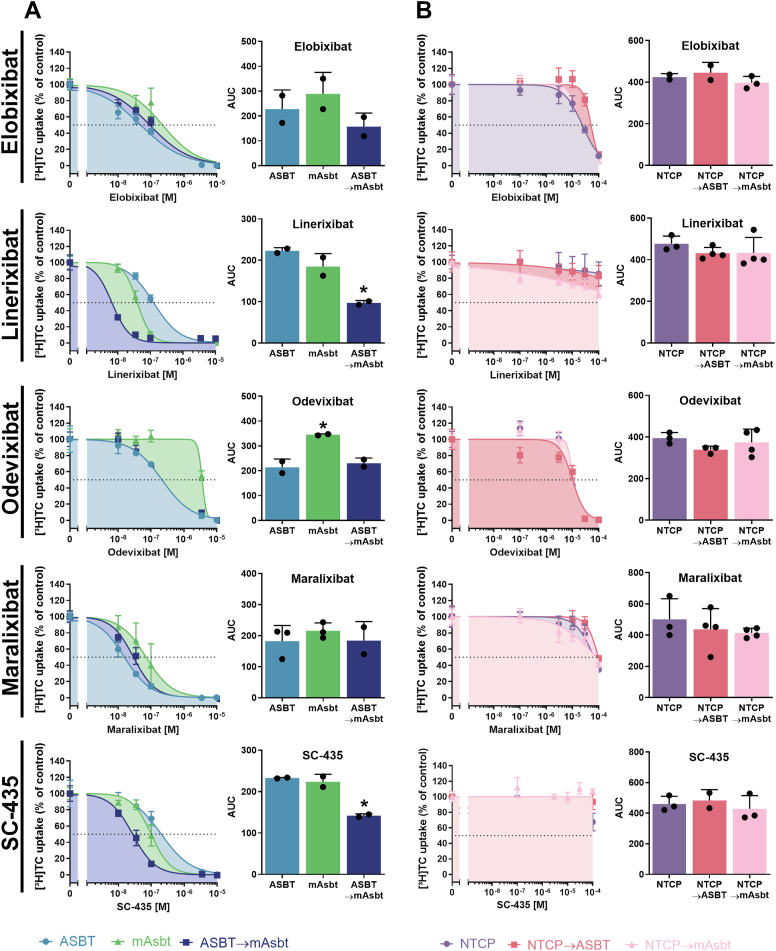
Bile acid transport inhibition of the ASBT and NTCP mutant constructs with the BARIs elobixibat, linerixibat, odevixibat, maralixibat and SC-435. Transport of 1 μM [^3^H]TC was analyzed for 10 min via the respective wild-type (ASBT, mAsbt, and NTCP) or mutant carriers (ASBT→mAsbt, NTCP→ASBT, NTCP→mAsbt) in the absence of any inhibitor (solvent control set to 100%) or in the presence of increasing concentrations of elobixibat, linerixibat, odevixibat, maralixibat, and SC-435, respectively. Transport data for [^3^H]TC in the absence of sodium were set to 0% for each carrier. Inhibition curves represent one out of at least two independent experiments each with quadruplicate determinations and all data points are shown as means ± SD. Inhibition curves were fit by non-linear regression analysis. The bar diagrams present area under the curve (AUC) values combined from at least two independent inhibition experiments, each with quadruplicate determinations. Transport inhibition data are separated for (A) ASBT, mAsbt, and the ASBT→mAsbt mutant, as well as for (B) NTCP, NTCP→ASBT, and NTCP→mAsbt. The x-axes of the IC_50_ diagrams are arranged in logarithmic representation. ∗Significantly different AUC values compared to the ASBT wild-type carrier according to one-way ANOVA with *P* < 0.05.

To analyze if linerixibat has different inhibition patterns for ASBT and mAsbt like 2164U90, [^3^H]TC transport studies were performed for both wild-type carriers at increasing concentrations of linerixibat. For this part of the study two independent experiments were performed for all carrier constructs and BARIs, each with quadruplicate determinations (n = 8). For all experiments, representative inhibition curves are shown in Figure 10. Due to the different slopes of the inhibition curves, the area under the inhibition curve (AUC) values from the combined transport inhibition data were used for statistical analysis instead of using the means of the two IC_50_ values. In the linerixibat inhibition studies, the inhibition curve of mAsbt was clearly shifted to the left compared to ASBT and, correspondingly, lower AUC values were determined for mAsbt compared to ASBT but without reaching the level of significance. Nevertheless, this trend confirms previous data measured for the linerixibat backbone compound 2164U90 (42). Interestingly, ASBT→mAsbt mutation significantly shifted the inhibition curve even more to the left and significantly reduced the AUC values compared to the wild-type ASBT construct (Fig. 10A). This data clearly indicates increased binding affinity of linerixibat after S294T/I295V double mutation of ASBT. Interestingly, this effect was also observed for the compound SC-435. In contrast, the transport inhibition patterns and AUC values between the wild-type and the ASBT→mAsbt mutant constructs were not different for elobixibat, odevixibat, and maralixibat. This indicates that the proposed ASBT 294/295 drug binding site is not uniformly involved in the binding of all BARIs. Of note, the wild-type ASBT and the ASBT→mAsbt mutant showed almost identical inhibition curves and AUC values for odevixibat. Interestingly, odevixibat was a much more potent inhibitor of the human ASBT protein compared to mAsbt, indicating contrary species specificities of linerixibat and odevixibat.

Although elobixibat, odevixibat, and maralixibat exhibited weak inhibition of wild-type NTCP, and linerixibat as well as SC-435 were completely inactive, it was of interest to determine whether mutating the amino acid residues 290 and 291 to the corresponding amino acids 294/295 of ASBT or mAsbt could enhance the inhibitory potency of any of the BARIs. Therefore, transport inhibition studies were also performed for the NTCP→ASBT and NTCP→mAsbt mutant constructs. As shown in Figure 10B, there were no differences between the mutants and wild-type NTCP for any of the compounds. For elobixibat, odevixibat and maralixibat, the inhibition curves and AUC values did not significantly change, and linerixibat and SC-435 were still inactive at both NTCP mutant constructs. This indicates that the 294/295 drug binding site of ASBT cannot simply be transferred to NTCP.

**Figure 11:**
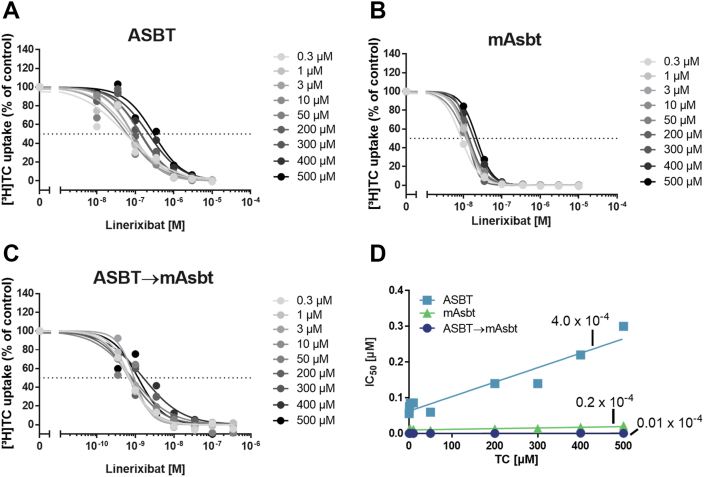
Bile acid transport inhibition at increasing substrate concentrations with linerixibat as the inhibitor. Transport experiments were performed in HEK293 cell lines stably expressing (A) ASBT, (B) mAsbt, or (C) the ASBT→mAsbt mutant, respectively, with [^3^H]TC at substrate concentrations ranging from 0.3 to 500 μM and respective increasing concentrations of linerixibat as inhibitor. Inhibition curves were calculated by non-linear regression analysis to obtain respective IC_50_ values. Data points represent means of quadruplicate determination. The x-axes of the IC_50_ diagrams are arranged in logarithmic representation. D: Correlation analysis between increasing TC substrate concentrations and the respective IC_50_ values for the wild-type carriers ASBT and mAsbt, as well as for the ASBT→mAsbt mutant. Slope calculation was done by linear regression analysis, and slope values are indicated in the figure for each carrier.

To analyze the possibility that the slightly different substrate affinities of ASBT, mAsbt, and ASBT→mAsbt with *K*_*m*_ values of 46.0 μM, 272.3 μM, and 25.9 μM, respectively, are responsible for the significant differences in the inhibitory potency of linerixibat, we analyzed the transport inhibition at all three carriers in more detail at different substrate concentrations ranging from 0.3 μM up to 500 μM. As shown in Figure 11A–C, linerixibat confirmed full transport inhibition even at the highest substrate concentration of 500 μM [^3^H]TC for all carriers. These experiments also confirmed the order of linerixibat inhibitor potency (ASBT→mAsbt > mAsbt > ASBT). However, whereas for ASBT the IC_50_ values for linerixibat inhibition increased with higher substrate concentrations with a slope of 4.0 × 10^−4^, the IC_50_ values remained quite stable for mAsbt and the ASBT→mAsbt mutant even at very high substrate concentrations. This data might indicate competitive replacement of linerixibat from ASBT at higher substrate concentrations and a less potent inhibition of ASBT, whereas the high-affinity binding and potent inhibition of mAsbt and ASBT→mAsbt by linerixibat could not be diminished by increased substrate concentrations. As in the ASBT→mAsbt mutant carrier, this shift was only achieved by S294T/I295V mutation; this position seems to play an important role for high-affinity binding of linerixibat and therefore seems to be part of the drug binding site.

## Discussion

This study provides the first direct, head-to-head comparison of elobixibat, linerixibat, maralixibat, and odevixibat regarding their inhibitory potencies against the intestinal BS transporter ASBT. In addition, potential cross-reactivity with the closely related transporters SOAT and NTCP, as well as with the key hepatic drug transporters OATP1B1, OATP1B3, and OATP2B1, was systematically evaluated. Moreover, the time-dependent inhibition profiles of these BARIs were characterized, and part of the binding site for linerixibat was identified through targeted mutagenesis studies.

### Head-to-head comparison of ASBT inhibition

IC_50_ values for ASBT inhibition have previously been reported for maralixibat, elobixibat, and odevixibat by the European Medicines Agency (EMA), the Food and Drug Administration (FDA), and the Pharmaceuticals and Medical Devices Agency (PMDA) in the context of drug approval (Table 2). However, apart from just the IC_50_ values, no detailed information about the experimental procedures was provided. Given that IC_50_ values can vary significantly depending on experimental conditions (*eg*, pH, temperature, solvent, probe substrate concentration, incubation time, compound solubility and stability, *etc.*), the expression system (cell line, amount of recombinant carrier protein overexpressed, potential co-factors, *etc.*), as well as data processing and IC_50_ calculation, IC_50_ values from different studies are often difficult to compare. On this background, the advantage of the present study is the direct head-to-head comparison of elobixibat, odevixibat, maralixibat, linerixibat, and SC-435 under the same experimental conditions, along with a detailed description of the experimental procedures. As summarized in Table 2, IC_50_ values for ASBT inhibition were previously reported as 0.53 nM for elobixibat (PMDA), 0.13 nM for odevixibat (EMA), and 0.28 nM for maralixibat (FDA), which are significantly lower than the IC_50_ values determined in the present study, namely 0.11 and 0.23 μM for elobixibat, 0.68 and 0.99 μM for odevixibat, and 0.14 and 0.22 μM for maralixibat (see Fig. 4). However, the direct comparison of the IC_50_ ratios for ASBT versus NTCP inhibition by elobixibat (0.0022 reported by the PMDA and 0.002-0.006 measured in this study) were in the same range, indicating that for direct compound comparison relative rather than absolute IC_50_ values should be considered. As a result of the present study, elobixibat, linerixibat, maralixibat, and odevixibat demonstrated comparable inhibitory potency toward ASBT, despite belonging to different chemical subclasses and differing significantly in their side-chain structures (see Fig. 2). Based on these findings, none of the compounds can be considered superior or inferior to the others regarding ASBT inhibition.

**Table 2:** Experimental transporter inhibition data of maralixibat, elobixibat, and odevixibat as reported by the EMA, the FDA, and the PMDA

Drug	Agency	Transporter inhibition and Therapeutic Drug Concentrations
Maralixibat (Livmarli)	EMA	Maralixibat is an OATP2B1 inhibitor based on in vitro studies. No potential for clinically relevant drug-drug interactions (DDI), but theoretical risk of interaction with substrates of this carrier.
		Reference: EPAR – Product Information Maralixibat, URL: https://www.ema.europa.eu/en/documents/product-information/livmarli-epar-product-information_en.pdf
		Maralixibat clinical drug concentrations in the 45 mg cohort study: C_max_ = 0.57 ng/ml (fed); 1.62 ng/ml (fasted).
		Reference: URL: https://www.ema.europa.eu/en/documents/variation-report/livmarli-h-c-005857-ii-0003-g-epar-assessment-report-variation_en.pdf
	FDA	In vitro, maralixibat did not inhibit the transporters OATP1B1 and OATP1B3.
		Maralixibat inhibits the drug transporter OATP2B1 in vitro with IC_50_ of 1.02 μM. This can potentially result in reduced absorption of drugs that rely on OATP2B1-mediated uptake in the gut.
		Maralixibat showed inhibition of ASBT expressed in baby hamster kidney H-14 cell line with IC_50_ of 0.28 nM.
		Maralixibat at 50 μM produced 5% inhibition of NTCP expressed in CHO cells.
		Reference: FDA – Approved Drugs – NDA 214662 Livmarli (maralixibat), URL: https://www.accessdata.fda.gov/drugsatfda_docs/nda/2021/214662Orig1s000IntegratedR.pdf
Elobixibat (Goofice)	PMDA	Elobixibat is an inhibitor of OATP1B1 with IC_50_ = 258 nM based on in vitro studies.
		Elobixibat is an inhibitor of OATP1B3 with IC_50_ > 600 nM based on in vitro studies. Elobixibat is unlikely to have inhibitory effects on OATP1B3 when used in clinical practice.
		Elobixibat inhibited ASBT with IC_50_ of 0.53 nM and NTCP with IC_50_ of 240 nM.
		Reference: Goofice, elobixibat hydrate, Report on the Deliberation Results, URL: https://www.pmda.go.jp/files/000232054.pdf
		Elobixibat clinical drug concentration in the 15 mg cohort study: C_max_ = 0.39 ng/ml.
		Reference: URL: https://www.pmda.go.jp/files/000232054.pdf
Odevixibat (Bylvay, Kayfanda)	EMA	Odevixibat does not inhibit OATP1B1 and OATP1B3.
		Reference: EPAR – Product Information, URL: https://www.ema.europa.eu/en/documents/product-information/bylvay-epar-product-information_en.pdf
		Odevixibat is a highly selective inhibitor of ASBT with an in vitro IC_50_ of 0.13 nM in ASBT-expressing HEK293 cells.
		Reference: EPAR – Risk management plan for odevixibat, URL: https://www.ema.europa.eu/en/documents/rmp/kayfanda-epar-risk-management-plan_en.pdf
	FDA	Odevixibat is an inhibitor of OATP1B1 and OATP1B3 with IC_50_ values of 0.308 μM and 0.697 μM, respectively, based on in vitro studies. This was considered as not clinically relevant.
		Reference: FDA-Approved Drugs – NDA 215498 BYLVAY (odevixibat), URL: https://www.accessdata.fda.gov/drugsatfda_docs/nda/2021/215498Orig1s000IntegratedR.pdf
		Odevixibat clinical drug concentration in the 120 μg/kg/day cohort study: C_max_ = 0.62 ng/ml.
		Reference: URL: https://www.ema.europa.eu/en/documents/product-information/bylvay-epar-product-information_en.pdf

All referred URLs were assessed on 26/06/2025.

### Cross-reactivities with SOAT and NTCP

Significant cross-reactivities of the analyzed BARIs with SOAT and, to some extent, also with NTCP were observed. This was not surprising given the close phylogenetic relationship among these carriers and findings from previous studies that also revealed significant inhibitor cross-reactivities among SLC10 carriers (22). ASBT, SOAT, and NTCP show quite high sequence homology and close overlap of the substrate and inhibitor patterns. Even if ASBT only transports BS and SOAT is specific for sulfated steroids, DHEAS has been identified as an inhibitor of ASBT, and BS are quite good inhibitors of SOAT (20, 22). NTCP has the broadest substrate spectrum and accepts both groups of molecules (BS and sulfated steroids) as substrates (Fig. 3). Moreover, many compounds have been identified as inhibitors of all three SLC10 carriers, *eg* some derivatives of the plant-derived triterpenoids betulin and betulinic acid, erythrosine B, BSP, or troglitazone (22, 35). More systematic comparative analyses of the ASBT and SOAT inhibition patterns revealed some compounds with potent inhibition of both carriers (*eg* the phenylsulfonylamino-benzanilide compound S1647, the dimeric bile acids S3068 and S1690, the thiobarbituric acid derivative S3740, or the propanolamine compound S8214), even if the 3D pharmacophore models of SOAT and ASBT do not clearly overlap (31, 43). A more recent study tried to identify structural requirements of inhibitors that would allow SLC10 carrier selectivity based on the phenylsulfonylamino-benzanilide compound S1647. In this study, systematic structure-activity relationships were analyzed for ASBT, NTCP, and SOAT using a set of more than 70 chemical derivatives. Consequently, all compounds exhibited varying degrees of cross-reactivity with one or more of the other carriers, making it impossible to achieve true selectivity for ASBT, NTCP, or SOAT (44).

All compounds, except of linerixibat, showed quite potent inhibition of the SOAT carrier with IC_50_ values ranging from 3-6 μM, being about one order of magnitude higher compared to ASBT inhibition. As this concentration range is not achieved in the bloodstream under clinical conditions (see Table 2), drug treatment with BARIs is unlikely to interfere with the physiological SOAT-mediated transport of 3′- and 17′-monosulfated steroid hormones such as dehydroepiandrosterone sulfate (DHEAS), esterone-3-sulfate (E_1_S), or testosterone sulfate (20, 22, 45). SOAT also plays a role in the cellular uptake of sulfated estrogen precursors such as E_1_S and so contributes to the proliferation of hormone-dependent breast cancer cells (46, 47). Based on this, derivatives of all three chemical classes (benzothiepines, benzothiadiazepines, and benzothiazepines) might be interesting pharmacological SOAT inhibitor candidates, even if the inhibitory potencies would need to be improved.

In the case of NTCP, BA transport inhibition was only detected for elobixibat, odevixibat, and maralixibat, however at very high inhibitor concentrations with IC_50_ ≥ 10 μM (Fig. 4). Even if drug concentrations are generally higher in the portal blood than in the systemic blood circulation it is very unlikely that the in vitro measured BARI cross-reactivities with NTCP have any significant effect on the hepatic BA transport in patients. In addition, interference of BARIs with the hepatitis B and D virus (HBV/HDV) entry receptor function of NTCP (48) is unlikely to occur in patients due to low potent in vitro NTCP inhibition. Virus attachment to NTCP occurs via the myristoylated preS1 domain (myr-preS1_2-48_ lipopeptide, here referred to as preS1 peptide) of the large virus envelope protein and represents the first essential step of HBV/HDV entry into hepatocytes (49). Based on this mechanism, pharmacological inhibition of preS1 peptide binding to NTCP is an attractive strategy for the development of HBV/HDV anti-viral drugs acting as virus entry inhibitors (50, 51, 52). Of note, several orally available small molecules have already been shown to inhibit NTCP and, thereby, prevent HBV/HDV infection of NTCP-expressing hepatoma cells. These include BA derivatives (*e.g.* DBA-41 (53) or obeticholic acid (54)), steroid-based compounds (*e.g.* ZINC000253533654 (55)), plant-derived compounds (*eg* vanitaracin A (56), exophilic acid (57), or betulinic acid (35)), or some FDA approved drugs (*eg* irbesartan (58), ritonavir (58), ezetimibe (59), cyclosporine A (60), rosiglitazone (61), or zafirlukast (61)). Most interesting for the context of the present study, a drug repurposing approach used several propanolamine derivatives that were derived from the development of BARIs at Sanofi and some of them showed significant NTCP inhibition and in vitro antiviral activity (*e.g.* S985852 (62)). In the present study, elobixibat and odevixibat inhibited preS1 binding to NTCP at high inhibitor concentrations. Based on this, repurposing of BARIs from the chemical classes of benzothiepines, benzothiadiazepines, and benzothiazepines would be an attractive approach to develop novel HBV/HDV entry inhibitors. This approach would require more detailed structure-activity relationship analyses to improve inhibitory potency and oral bioavailability by molecular drug design.

### Cross-reactivity with hepatic OATPs

The inhibition of OATP carriers by the analyzed BARIs was likewise anticipated, considering their role as multidrug transporters (63). Of note, these OATP carriers share some substrates (*e.g.* BS, DHEAS, E_1_S, and rosuvastatin) and inhibitors (*e.g.* BSP, cyclosporine A, and troglitazone) with the SLC10 carriers (36). Based on this it was not surprising that elobixibat, linerixibat, odevixibat, maralixibat, and SC-435 all showed inhibition of OATP1B1, OATP2B1, and OATP1B3. More detailed analysis in the present study revealed equipotent inhibition of all three carriers with elobixibat and odevixibat with IC_50_ of 2-5 μM and slightly lower inhibitory potency for maralixibat with IC_50_ of 5-9 μM. In contrast, linerixibat and SC-435 were only potent inhibitors of OATP1B3 with IC_50_ of 2-8 μM and both compounds were less active at OATP1B1 and OATP2B1 with IC_50_ of >10 μM. As the hepatic OATP carriers are important drug transporters of the liver (63), potent pan-inhibition of these carriers is at risk to affect the hepatobiliary elimination and pharmacokinetic behavior of dominantly OATP transported drugs (36). Therefore, new drug candidates are typically analyzed for potential cross-reactivity with the hepatic OATPs (64). This was also the case for the approved drugs elobixibat, odevixibat, and maralixibat (see Table 2). However, the results of this screening were not published in research papers but are just listed in the respective product information of the EMA, the FDA, and the PMDA (see Table 2). In contrast, the present study provides systematic testing of all BARIs head-to-head against OATP1B1, OATP2B1, and OATP1B3.

Maralixibat has previously been reported as inhibitor of OATP2B1 with IC_50_ of 1.02 μM (Table 2). The IC_50_ values of 6.0 and 7.5 μM measured for OATP2B1 in the present study were somewhat higher. But in contrast to what was reported by the FDA, maralixibat also inhibited OATP1B1 and OATP1B3 in the present study with IC_50_ values of 8.3 and 9.0 μM, as well as 5.2 and 5.5 μM, respectively (Fig. 6). Conflicting data has been reported for odevixibat. While the FDA states that odevixibat inhibits OATP1B1 and OATP1B3 with IC_50_ values of 0.3 μM and 0.7 μM, respectively, the EMA reports that odevixibat does not inhibit OATP1B1 and OATP1B3. No data was available regarding OATP2B1 inhibition by odevixibat. The present study measured somewhat higher IC_50_ values of 2-5 μM for all three carriers (Fig. 6). For elobixibat, IC_50_ values of 258 nM and >600 nM have been reported for OATP1B1 and OATP1B3 inhibition, respectively; however, no data was available for OATP2B1 (Table 2). The present study revealed IC_50_ of 4-5 μM for all three OATP carriers. Considering the low oral bioavailability of maralixibat, elobixibat, and odevixibat, with clinical drug concentrations in the low nanomolar range (EMA, FDA, PMDA, Table 2) compared with the relatively high IC_50_ values for OATP inhibition in the micromolar range, potential DDI at the OATP carriers are not considered as clinically relevant.

Currently, there are no approved drugs containing linerixibat. However, this compound is presently tested in two phase III clinical trials for the treatment of cholestasis and pruritus in patients with primary biliary cholangitis. In a recent study by Zamek-Gliszczynski *et al.* (2021) the following cross-reactivities with OATP carriers were reported: IC_50_ of 2.69 μM for OATP1B1 and 0.265 μM for OATP1B3 (65). In addition, it was mentioned that linerixibat is a substrate of the OATP1B1 and OATP1B3 carriers. As also observed for elobixibat and odevixibat, the reported IC_50_ values are lower compared to the present study (here being 12.0 and 29.1 μM for OATP1B1, as well as 2.2 and 3.6 μM for OATP1B3). In addition, the present study determined the IC_50_ for linerixibat at OATP2B1 to 22.5 and 26.1 μM, while it was previously reported that linerixibat is not a substrate of this transporter (65). As linerixibat, like elobixibat, odevixibat, and maralixibat was intentionally designed for low intestinal absorption (66) systemic exposure and the potential risk for DDIs in the clinical context are minimal.

### Differences in the time-dependent ASBT inhibition Pattern

Even if the substrate and inhibition patterns of ASBT, NTCP, and SOAT have been intensively investigated in the last two decades (22, 67, 68), the exact transport and inhibition modes are not completely understood so far. Crystal structures from two bacterial SLC10 homologues, namely Asbt_Nm_ from *Neisseria meningitidis* (37) and Asbt_Yf_ from *Yersinia frederiksenii* (38) revealed two inverted structural repeats of five TMD that are organized in a core structure (composed of TMD 3–5 and 8–10) and a panel structure (composed of TMD 1, 2, 6, and 7). Based on these structures, an alternating-access transport mechanism has been proposed with substrate binding to an outward-facing conformation and intracellular substrate release from an inward-facing conformation (38). Accordingly, the transporter protein must switch between outward- and inward-facing states to allow substrate transport and sodium ions might control this conformational transition (69). However, sequence homology of these bacterial proteins to the human SLC10 carriers ASBT, NTCP, and SOAT is quite low (at 25%) and even if the naming would suggest it, there is no clear homology to ASBT. Finally, these bacterial proteins have one additional N-terminal TMD that is not present in the human ASBT, NTCP, and SOAT structures.

More recently, four independent cryo-EM structures have been resolved for human NTCP covering slightly different conformations (26, 27, 40, 41). All structures are quite similar to the bacterial structures of Asbt_Nm_ and Asbt_Yf_, however, NTCP has only 9 TMD with the core structure composed of TMD 2–4 and 7–9 and the panel structure composed of TMD 1, 5, and 6. Surprisingly, two of these structures were captured in an open pore-like conformation (PDB 7PQQ (40) and PDB 7ZYI (26)). Most interesting for the question of the substrate transport mode is the substrate-bound conformation resolved by the group of Kaspar Locher (26), revealing a tunnel-like structure through the protein that allows access from the extracellular and intracellular site. Within this tunnel structure two distinct substrate binding sites have been identified and were named as S_out_ and S_in_. Substrate binding to S_out_ that is accessible from the extracellular side might plug the tunnel, thereby preventing Na^+^ ion leakage. Subsequently, the BS molecule might be shifted from S_out_ to S_in_, while the transporter is reloading one additional BS molecule to S_out_ from the outside. BS molecules then can only be released to the intracellular site from S_in_, probably involving co-release of two Na^+^ ions. In this scenario, BS binding to S_out_ is still accessible from the extracellular milieu and would be important to seal the transport tunnel (26).

According to this transport model, inhibitors might behave like the BS substrates and initially bind to S_out_ before they reach S_in_. Interestingly, the present study identified three distinct inhibition pattern among the analyzed BARIs. Odevixibat and maralixibat exhibited long-lasting, full ASBT inhibition even after washout and in the absence of inhibitor in the medium, a phenomenon categorized as inhibitor-decoupled inhibition. Based on the structural information mentioned earlier, we propose that odevixibat and maralixibat may behave like BS substrate by initially binding to S_out_ and subsequently to S_in_. Unlike BS substrates, these inhibitors might then remain bound to S_in_ without being released into the intracellular milieu, so effectively clogging the transport pore. While in this scenario during the washout phase the inhibitor bound at S_out_ might be removed, the inhibitor molecule bound to S_in_ might remain in place without leaving the pore to either the extracellular or the intracellular side. As an alternative explanation, differences in the dissociation (off-) rates of the inhibitors may account for the observed effects. Accordingly, odevixibat and maralixibat may just remain tightly bound to their respective binding sites for longer periods. Finally, it also cannot be excluded that the ASBT protein may be destabilized upon inhibitor binding or that it undergoes endocytosis.

In contrast to odevixibat and maralixibat, linerixibat demonstrated full ASBT inhibition with rapid and complete recovery in the absence of inhibitor in the medium, indicative of inhibitor-coupled inhibition. This could be explained by linerixibat binding solely to S_out_ from where it can easily be released and replaced by BS molecules. Finally, the intermediate inhibition pattern of elobixibat, with partial recovery in the absence of inhibitor in the medium, could be attributed to high-affinity binding to S_out_ but low-affinity binding to S_in_. This allows for recovery of some transport activity during washout, while some ASBT molecules remain inhibited by elobixibat bound to S_in_. This proposed mode of inhibition requires further investigation, including clinical data that might reveal differences in the coupling of the pharmacokinetic and pharmacodynamic profiles of the various BARIs. In this context, the inhibitor-decoupled inhibition shown by odevixibat and maralixibat might be advantageous due to their long-lasting ASBT inhibition, even at low or absent inhibitor concentrations in the gut lumen.

### Localization of part of the linerixibat binding site at ASBT

In a previous study, Hallén *et al.* (2002) demonstrated that the benzothiazepine-based inhibitor 2164U90 is a much better inhibitor of mAsbt (*K*_*i*_ = 0.068 μM) than of ASBT (*K*_*i*_ = 10 μM) (42). This 150-fold difference in inhibitor potency is interesting because ASBT and mAsbt differ at only a few amino acid positions, namely at the N- and C-terminal ends that most likely are not involved in substrate and inhibitor binding, as well as at 41 individual amino acid residues in the transmembrane part of the protein (TMD 1 – TMD 9), including S294/I295 in human ASBT and T294/V295 in mAsbt. By a series of mutation experiments, Hallén *et al.* showed that this position is responsible for the different inhibition kinetics of the ASBT and mAsbt proteins, suggesting that this domain is part of the inhibitor binding pocket or is even directly involved in 2164U90 binding (42). In this study, the *K*_*m*_ values for [^14^C]TC transport were determined to 9.4 μM for ASBT and 4.3 μM for mAsbt using substrate concentrations ranging from 5-40 μM. These values clearly differ from the *K*_*m*_ values measured in the present study using [^3^H]TC at 1–200 μM, namely *K*_*m*_ = 46.0 μM for ASBT and *K*_*m*_ = 272.3 μM for mAsbt.

The present study could identify more potent inhibition of mAsbt compared to human ASBT for the 2164U90 derivative linerixibat. This suggests that the above-mentioned S294/I295 inhibitor binding site of ASBT and T294/V295 in mAsbt might play a role for linerixibat binding. This hypothesis was supported by the fact that linerixibat is structurally very similar to 2164U90. It was demonstrated that ASBT by S294T and I295V mutation (ASBT→mAsbt) was more potently inhibited by linerixibat, suggesting that this domain does also play a role for linerixibat binding. Even if the transport rate for [^3^H]TC of the ASBT→mAsbt mutant was significantly lower than the transport rates of ASBT and mAsbt, the substrate affinity was not significantly affected (*K*_*m*_ = 46.0 μM for ASBT and *K*_*m*_ = 25.9 μM for the ASBT→mAsbt mutant). Furthermore, ASBT and the ASBT→mAsbt mutant were inhibited nearly identical by elobixibat, odevixibat, and maralixibat. Moreover, by using a broad range of TC substrate concentrations (0.3–500 μM), it was clearly shown that the IC_50_ values for linerixibat remained nearly stable for mAsbt and the ASBT→mAsbt mutant, confirming high affinity binding of linerixibat. In contrast, linerixibat IC_50_ values increased at high substrate concentrations for ASBT, most likely by competitive inhibitor replacement. Based on these data, it can be concluded that the amino acid residues S294 and I295 of ASBT are involved in linerixibat binding. However, this does not appear to be the case for elobixibat, odevixibat, and maralixibat, leaving the inhibitor binding sites of these inhibitors unresolved and in need of further investigation.

In conclusion, the clinically used ASBT inhibitors elobixibat, odevixibat, and maralixibat demonstrate potent cross-reactivity with SOAT and NTCP. In contrast, linerixibat was ASBT-specific and was the only BARI for which the S294/I295 site of ASBT was relevant for inhibitor binding. Additionally, while linerixibat readily accessed its inhibitor binding site, odevixibat and maralixibat exhibited an inhibitor-decoupled mode of inhibition. The clinical relevance of the *in vitro*-described BARI cross-reactivities is limited due to their low oral bioavailability. However, the clinical significance of the different inhibition pattern should be further investigated, particularly regarding the potential decoupling of the pharmacodynamic properties of odevixibat and maralixibat from their pharmacokinetic profiles, as suggested in the present study.

## Data availability

All data are contained within the manuscript.

## Conflict of interests

The authors declare the following financial interests/personal relationships which may be considered as potential competing interests:

Yohannes Hagos is CEO of PortaCellTec Biosciences GmbH, a company providing commercial transport assays. Joachim Geyer declares consulting activities for Albireo Pharma.
